# P-1692. Evaluation of Antibiotic Appropriateness at Discharge in a Community Teaching Hospital

**DOI:** 10.1093/ofid/ofae631.1858

**Published:** 2025-01-29

**Authors:** Hinal Patel, Melissa Poulsen

**Affiliations:** Penn Medicine Princeton Medical Center, Plainsboro, New Jersey; Penn Medicine Princeton Medical Center, Plainsboro, New Jersey

## Abstract

**Background:**

Antimicrobial therapy is prescribed for approximately 20% of patients upon hospital discharge. The CDC and Joint Commission emphasize antimicrobial stewardship as a standard of care. While inpatient programs have processes in place to monitor and optimize antimicrobial therapy, monitoring and intervention during transitions of care is an unmet need for many hospital-based antimicrobial stewardship programs. We sought to evaluate antibiotic use at discharge at our institution to help develop potential strategies for monitoring and intervention.

Figure 1

**Figure:**
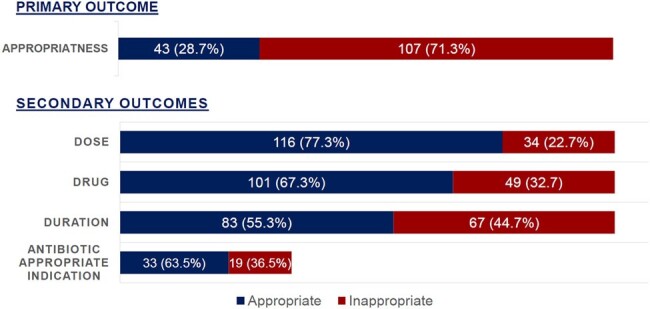

Appropriateness of overall discharge antimicrobial therapy and breakdown by specific domains

**Methods:**

This retrospective single center chart review evaluated adult patients discharged on oral antibiotics and diagnosed with pneumonia, skin and soft tissue infection, urinary tract infection, intra-abdominal infection, or bacteremia secondary to these infections. Exclusion criteria included pharmacist intervention on discharge orders, discharge parenteral antibiotics, and immunocompromised patients. The primary outcome was antibiotic appropriateness defined as right drug, dose, duration, and antibiotic-appropriate indication. Secondary outcomes included total duration of antibiotics, 30-day mortality, infection recurrence, and Clostridium difficile occurrence. Descriptive statistics were used for demographics and primary and secondary endpoints, and comparative statistics were used to evaluate the relationship between secondary outcomes and appropriateness.

**Results:**

A total of 150 patients were included, of which 43/150 (28.7%) were prescribed appropriate antimicrobial therapy on discharge. Cefpodoxime was the most commonly prescribed antibiotic (45/150, 30%). The most common cause of inappropriate therapy was excess duration. The average excess duration of therapy was 4.4 days. The most common antibiotic indication was urinary tract infection, of which 33/52 (63.5%) cases were asymptomatic. Of note, the inappropriate use group had a statistically significant higher rate of 30-day readmission (12/107 versus 0/43, p = 0.022).

**Conclusion:**

Inappropriate discharge antibiotics were common and associated with increased 30-day readmission rates, indicating a need for increased antimicrobial stewardship monitoring and intervention on hospital discharge.

**Disclosures:**

**Hinal Patel, PharmD, BCPS, BCIDP**, Abbvie: Stocks/Bonds (Public Company)

